# Prevalence of pathological FFR_CT_ values without coronary artery stenosis in an asymptomatic marathon runner cohort

**DOI:** 10.1007/s00330-021-08027-0

**Published:** 2021-05-26

**Authors:** Sebastian Gassenmaier, Ilias Tsiflikas, Simon Greulich, Jens Kuebler, Florian Hagen, Konstantin Nikolaou, Andreas M. Niess, Christof Burgstahler, Patrick Krumm

**Affiliations:** 1 Department of Diagnostic and Interventional Radiology, University of Tuebingen, Tübingen, Germany; 2 Department of Cardiology and Angiology, University of Tuebingen, Tübingen, Germany; 3 Department of Internal Medicine V, Sports Medicine, University of Tuebingen, Hoppe-Seyler-Straße 6, 72076 Tübingen, Germany

**Keywords:** Fractional flow reserve, Myocardial, Computed tomography angiography, Coronary artery disease, Running

## Abstract

**Objectives:**

To evaluate computed tomography fractional flow reserve (FFR_CT_) values in distal parts of the coronaries in an asymptomatic cohort of marathon runners without any coronary stenosis for potentially false-positive values.

**Methods:**

Ninety-eight asymptomatic male marathon runners (age 53 ± 7 years) were enrolled in a prospective monocentric study and underwent coronary computed tomography angiography (CCTA). CCTA data were analyzed for visual coronary artery stenosis. FFR_CT_ was evaluated in 59 participants without coronary artery stenosis in proximal, mid, and distal coronary sections using an on-site software prototype.

**Results:**

In participants without coronary artery stenosis, abnormal FFR_CT_ values ≤ 0.8 in distal segments were found in 22 participants (37%); in 19 participants in the LAD; in 5 participants in the LCX; and in 4 participants in the RCA. Vessel diameters in participants with FFR_CT_ values > 0.80 compared to ≤ 0.80 were 1.6 ± 0.3 mm versus 1.5 ± 0.3 mm for distal LAD (*p = *0.025), 1.8 ± 0.3 mm versus 1.6 ± 0.5 mm for distal LCX (*p* = 0.183), and 2.0 ± 0.4 mm versus 1.5 ± 0.2 mm for distal RCA (*p* < 0.001).

**Conclusions:**

Abnormal FFR_CT_ values of ≤ 0.8 frequently occurred in distal coronary segments in subjects without any anatomical coronary artery stenosis. This effect is only to some degree explainable by small distal vessel diameters. Therefore, the validity of hemodynamic relevance evaluation using FFR_CT_ in distal coronary artery segment stenosis is reduced.

**Key Points:**

*• Abnormal FFR*_*CT*_ *values (≤ 0.8) occurred in over a third of the subjects in the distal LAD despite the absence of coronary artery stenosis..*

*• Therefore, the validity of hemodynamic relevance evaluation in distal coronary artery segment stenosis is reduced.*

*• Decision-making based on abnormal FFR*_*CT*_ *values in distal vessel sections should be performed with caution and only in combination with visual assessment of the grade of stenosis..*

**Supplementary Information:**

The online version contains supplementary material available at 10.1007/s00330-021-08027-0.

## Introduction

One of the most important developments in the diagnosis of significant coronary artery disease (CAD) over the last two decades is the assessment of the hemodynamic significance of coronary artery stenosis using fractional flow reserve (FFR) and systematic evaluation of its clinical value. It could be shown that an FFR value below 0.75–0.80 defines a hemodynamically significant stenosis that should be treated by revascularization [1–4]. However, a drawback of this method is its invasiveness. Here, a noninvasive approach to detect coronary stenosis is the emerging technique of coronary computed tomography angiography (CCTA). Meanwhile, CCTA has been developed as a well-established and cost-effective imaging modality for the evaluation of CAD, especially to exclude obstructive stenosis due to its high negative predictive value [5–8]. Technical progress and the introduction of various optimized acquisition techniques and strategies allowed considerable radiation dose reduction—one of the most significant issues at the beginning—to sub-millisievert levels [9]. Recently, the development of post-processing procedures enabled the calculation of computed tomography fractional flow reserve (FFR_CT_) values based on CCTA datasets using computational fluid dynamics or machine learning algorithms. Although good diagnostic accuracy of FFR_CT_ has been reported, a major issue remains the evaluation of the distal parts of the vessels, with a potentially limited specificity [10]. Due to physiological tapering of the coronary vessels, FFR_CT_ evaluation may yield values below 0.8 resulting in false-positive results.

However, the exact prevalence of this finding is unknown, especially in asymptomatic persons without signs of CAD who perform regular exercise which is known to have a positive impact on endothelial function. Therefore, the aim of this study was to evaluate FFR_CT_ in a collective of asymptomatic marathon runners without CAD who prospectively underwent CCTA for CAD screening purposes.

## Materials and methods

### Ethics approval

This prospective study was approved by the institutional review board (processing number 158/2011B01) and the German Federal Office for Radiation Protection (processing number Z5-22462/2-2011-22). All participants gave informed consent to participate in this investigation. The study was in line with the declaration of Helsinki.

### Study design

Ninety-eight asymptomatic male marathon runners above 45 years of age were prospectively recruited between 2012 and 2014 for CAD screening and estimation of risk for sudden cardiac death in this cohort. Prior to the CCTA examination, all participants underwent a dedicated screening protocol to exclude unknown cardiovascular disease or other severe illness. This screening involved physical examination, resting ECG, and echocardiography. All participants additionally performed a treadmill stress test for maximal oxygen uptake evaluation. Blood samples were taken after 8 h of fasting to determine blood lipid levels (total cholesterol, LDL, HDL, triglyceride).

Participants with known CAD, known allergies to iodinated contrast agents, impaired renal function (glomerular filtration rate < 60 mL/min/1.73 m²), or hyperthyroidism were excluded from this study. Clinical data and visual CCTA results of all study subjects have been published previously [11, 12].

### Coronary computed tomography angiography

All examinations were performed using a modern dual-source CT scanner (Siemens Somatom Definition Flash; Siemens Healthineers). After scout acquisition in a supine position, non-contrast high-pitch ECG triggered calcium scoring was acquired in cranio-caudal scanning direction using the following parameters: collimation 2 × 64 × 0.6 mm with a *z*-axis flying focal spot, gantry rotation 280 ms, pitch 3.4, tube current of 70 mA per rotation applying automatic tube current modulation, tube voltage of 120 kV. For the calculation of the systemic circulation time, a non-ionic iodinated contrast agent bolus of 10 mL (370 mg iodine/mL, Ultravist 370, Bayer Healthcare) followed by a saline flush of 20 mL with a flow of 6 mL/s was applied using a dual-head-injector (CT Stellant, Medrad). For CCTA, a contrast agent dose of 70 mL followed by a saline flush was then applied with the same flow parameters. Depending on the heart rate, participants underwent high-pitch (≤ 60 beats per minute (bpm)) or prospective sequential step-and-shoot (> 60 bpm) acquisition at 60% of the R-R-interval. Technical parameters for high-pitch acquisition were as follows: collimation 2 × 64 × 0.6 mm with a *z*-axis flying focal spot, gantry rotation 280 ms, pitch 3.4, tube current of 350mA per rotation with automatic tube current modulation, tube voltage of 100 kV. The step-and-shot acquisition protocol was already previously described [11].

Images were reconstructed using a 3-mm slice thickness for calcium scoring (B35f) and 0.75 mm slice thickness for CCTA (B26f).

### CCTA and FFR_CT_ evaluation

CCTA datasets were evaluated by two experienced radiologists in consensus via visual analysis using thin slab maximum intensity projections and curved multiplanar reconstructions. FFR_CT_ was determined using an on-site software prototype (cFFR 3.2, syngo.via Frontier, Siemens Healthineers) as previously described [13]. FFR_CT_ values were determined using the reporting system of the American Heart Association [14]: left main (LM, segment 5), left descending artery (LAD) proximal (segment 6), mid (segment 7), and distal (segment 8); left circumflex artery (LCX) proximal (segment 11), mid (border of segments 11 and 13), and distal (segment 13); and right coronary artery (RCA) proximal (segment 1), mid (segment 2), and distal (segment 3). No side branches were evaluated.

FFR_CT_ values ≤ 0.8 were regarded as significant stenosis. In visual analysis, the degree of maximal stenosis was categorized according to the CAD-RADS scheme: no-stenosis (CAD-RADS 0), non-significant stenosis (1–49% stenosis; CAD-RADS 1 and 2), obstructive stenosis (50–99%; CAD-RADS 3 and 4), and total occlusion (CAD-RADS 5) [15]. According to the degree of stenosis, participants were allocated to three groups: (I) no coronary artery stenosis, (II) non-significant coronary artery stenosis (< 50%), and (III) obstructive coronary artery stenosis (≥ 50%).

### Statistical analysis

Proprietary statistical software was used for evaluation (MedCalc Statistical Software version 18.10; MedCalc Software bvba). The Mann-Whitney *U* test and the Kruskal-Wallis test were used for comparison between the three stenosis groups. The Fisher-Freeman-Halton test for frequency of occurrence of pathologically low FFR_CT_ values ≤ 0.8 between the different groups of coronary artery dominance for each coronary artery respectively was calculated with SPSS 27 (IBM Corp.). The significance level alpha was set at 0.05.

## Results

### Subjects’ characteristics

Ninety-eight participants underwent CCTA. The mean participants’ age was 53 ± 7 years and all participants were male. The median personal best marathon time was 3:28 h with an interquartile range of 41 min. The mean Framingham risk score was 5.8 ± 3.5. In 31 runners, coronary artery stenosis was present in visual CCTA evaluation. Non-significant coronary artery stenosis was found in 22 subjects in the LAD, 5 subjects in the LCX, and 8 subjects in the RCA. In total, 23 subjects were affected with non-significant coronary artery stenosis. Obstructive stenosis ≥ 50% was seen in a total of 9 vessels (LAD: *n* = 6; LCX: *n*=2; RCA: *n* = 1) in 8 participants. In four of these eight participants, a stenosis of ≥ 70% (CAD-RADS 4) was found (maximum stenosis 80%). Further clinical data is presented in Table 1. The mean volume computed tomography dose index (CTDI_vol_) for CCTA was 6.0 ± 3.0 mGy. Further technical parameters are shown in Table 2.

**Table 1:** Characteristics of the study group

Characteristics	Values
Subjects	*n* = 98 (90 with successfull FFR_CT_)
Subjects’ characteristics	
Age Height Bodyweight BMI Body fat Systolic blood pressure Diastolic blood pressure Personal best marathon time (median; IQR) Number of marathons (median; IQR) VO2 max Agatston calcium score (median; IQR)	53 ± 7 years (range: 45–74 years) 179.7 ± 5.6 cm 76.6 ± 8.9 kg 23.7 ± 2.3 13.3 ± 5.5 % 134 ± 17 mmHg 85 ± 9 mmHg 3:28 h (41 min) 10 (16) 48.0 ± 6.0 mL/kg/min 0 (26)
Blood lipid and glucose levels	
Triglyceride LDL HDL Cholesterol Glucose levels	94 ± 57 mg/dL 107 ± 28 mg/dL 62 ± 13 mg/dL 201 ± 34 mg/dL 96 ± 9 mg/dL
Risk scores and medication	
Framingham score Cardiac-related medication Beta-blocker ACE inhibitor / AT1 antagonist Diuretics Statin Ca-antagonist	5.8 ± 3.5 *n* = 9 *n* = 1 *n* = 7 *n* = 0 *n* = 3 *n* = 2
Coronary artery stenosis	
Coronary artery stenosis Obstructive coronary artery stenosis (≥ 50%) Coronary vessels with obstructive stenosis (≥ 50%)	*n* = 31 *n* = 8 *n* = 9
Coronary dominance in participants without coronary artery stenosis (*n* = 59)	
Left dominant Right dominant Codominant	*n* = 7 *n* = 20 *n* = 32

Abbreviations: *IQR*, interquartile range

**Table 2:** Calcium scoring and CCTA radiation dose parameters

Characteristics	Values
Calcium scoring	
Heart rate CTDI_vol_ DLP Effective dose	60 ± 10 bpm 0.9 ± 0.2 mGy 19.5 ± 3.9 mGy*cm 0.3 ± 0.1 mSv
CCTA	
High-pitch / sequential acquisition Heart rate CTDI_vol_ DLP Effective dose	N = 34 (38%) / 56 (62%) 59 ± 10 bpm 6.0 ± 3.0 mGy 89.6 ± 35.3 mGy*cm 1.3 ± 0.5 mSv

Abbreviations: *CTDI*_*vol*_, volume computed tomography dose index; *DLP*, dose length product

**Fig. 1:**
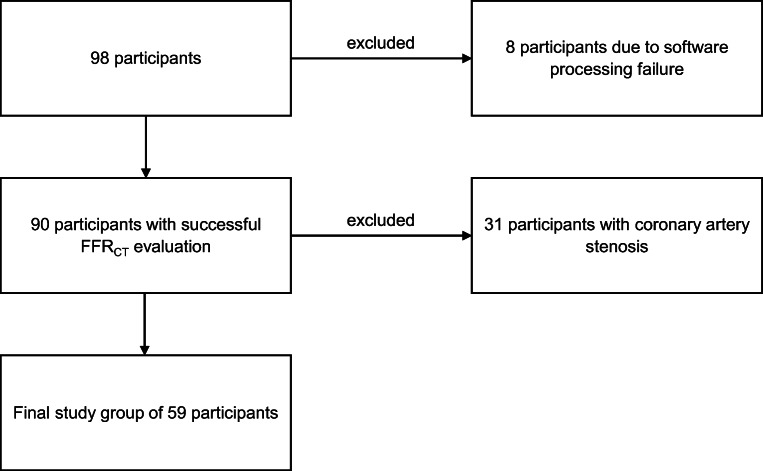
The flowchart of this study

Ninety datasets could successfully be evaluated using the FFR_CT_ prototype. Eight datasets were unevaluable for the FFR_CT_ prototype software due to prototype software coronary artery segmentation processing failure. Thirty-one participants showed signs of coronary artery stenosis and were excluded from the main evaluation (Fig. 1). Data and analysis of the excluded 31 participants can be found in the electronic supplementary material (ESM).

### Analysis of participants without coronary artery stenosis

Fifty-nine participants showed no signs of coronary artery stenosis in any vessel. No participant showed FFR_CT_ values below 0.8 in the proximal LAD, LCX, or RCA.

Overall, abnormal FFR_CT_ values in distal segments were found in 22 participants (37%): FFR_CT_ values ≤ 0.8 were found in two participants in the mid LAD, in two participants in the mid LCX, in 19 participants in the distal LAD, in five participants in the distal LCX, and in four participants in the distal RCA. The detailed FFR_CT_ values can be found in Table 3.

**Table 3:** Per vessel analysis: computed tomography fractional flow reserve (FFR_CT_) results with number and percentage of vessels with FFR ≤ 0.8 in parentheses

	Proximal	Mid	Distal
Vessels in subjects without coronary artery stenosis in any vessel (*n* = 59)			
LAD (*n* = 59)	0.98 ± 0.02 (0; 0%)	0.93 ± 0.05 (2; 3%)	0.81 ± 0.10 (19; 32%)
LCX (*n* = 59)	0.99 ± 0.02 (0; 0%)	0.94 ± 0.06 (2; 3%)	0.89 ± 0.07 (5; 8%)
RCA (*n* = 59)	0.99 ± 0.01 (0; 0%)	0.96 ± 0.02 (0; 0%)	0.89 ± 0.07 (4; 7%)

Vessel diameters in the distal LAD were significantly larger between participants with FFR_CT_ values > 0.80 (1.6 ± 0.3 mm) compared to participants with FFR_CT_ values ≤ 0.80 (1.4 ± 0.3 mm; *p* = 0.040). The vessel diameter evaluation of the distal LCX showed no significant difference with 1.8 ± 0.3 mm (FFR_CT_ > 0.80) versus 1.5 ± 0.5 mm (FFR_CT_ ≤ 0.80; *p* = 0.285). Vessel diameters of the distal RCA were significantly larger with 2.0 ± 0.5 mm in participants with FFR_CT_ > 0.80 compared to 1.4 ± 0.1 mm in participants with FFR_CT_ ≤ 0.80 (*p* = 0.007).

Coronary dominance had no significant impact on the occurrence of pathologically low FFR_CT_ values in distal coronary artery segments: Frequency of occurrence of pathologically low FFR_CT_ values ≤ 0.8 between the different groups of coronary artery dominance for each distal coronary artery respectively was not significant; Fisher-Freeman-Halton test for LAD: *p* = 0.227; LCX: *p* = 0.183; RCA: *p* = 0.170. Detailed evaluation, see Table 7 in the ESM.

**Fig. 2:**
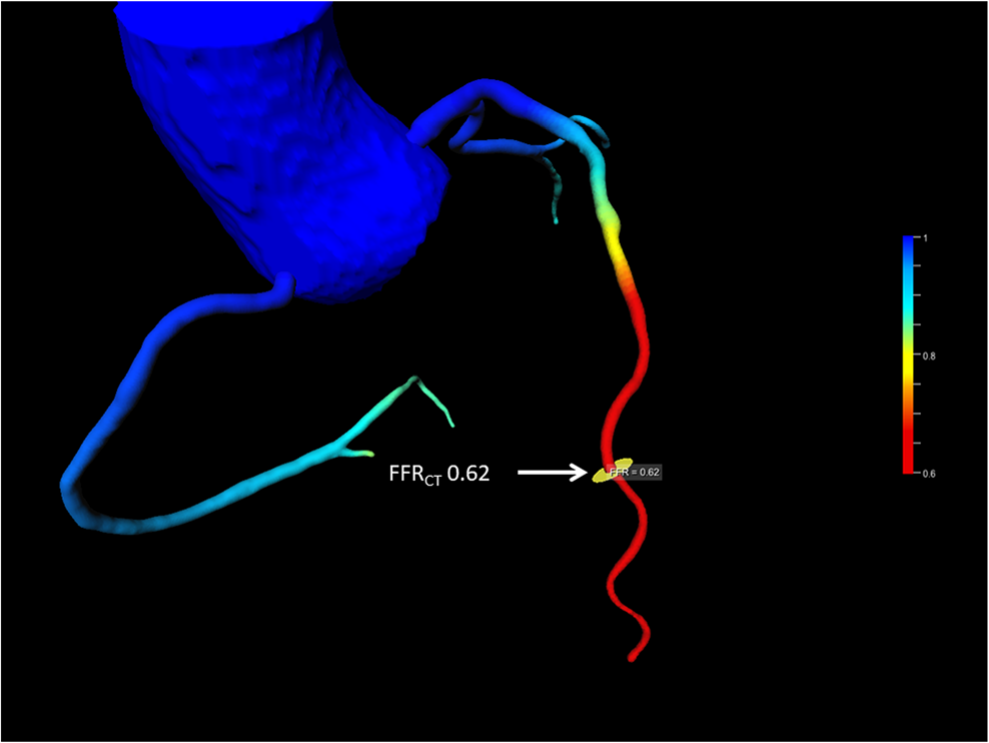
Example of FFR_CT_ evaluation without coronary stenosis. Despite the absence of coronary stenosis, the FFR_CT_ evaluation resulted in values of ≤ 0.8 in the distal section. The location of FFR_CT_ measurement is marked by an arrow. *Abbreviations: FFR*_*CT*_, CT fractional flow reserve

Figure 2 shows an example of FFR_CT_ evaluation in a participant without stenosis.

## Discussion

This study demonstrated that in an asymptomatic cohort of male marathon runners without clinical or visually assessable coronary artery stenosis, FFR_CT_ evaluation results in abnormal FFR_CT_ values (FFR_CT_ ≤ 0.8) in distal segments of coronary arteries in over one-third of subjects. This effect is independent from coronary artery dominance and only to some degree explainable by small distal vessel diameters. Isolated vessel diameter is one important but not the only factor influencing FFR_CT_. This finding limits the use of the method in distal vessel segments.

This is the first systematic FFR_CT_ evaluation in an asymptomatic cohort of male marathon runners initially prospectively recruited at that time. Correct evaluation and grading of FFR_CT_ values are of utmost importance for the estimation of relevant coronary artery stenosis, since the appropriate use of CCTA has been reported to decrease, and not to increase the number of unnecessary invasive coronary angiographies [16]. CCTA alone can be considered an established diagnostic modality in patients with low or intermediate pre-test probability for the exclusion of CAD [17]. It was previously shown that FFR_CT_ can successfully be used in an acute chest pain setting for the decision or deferral of invasive angiography [18]. It was also shown that FFR_CT_ provides reliable results compared to invasive catheter-based measurements [10, 19–24]. In a recent publication, it was demonstrated that also on-site FFR_CT_ might be able to change patient management and improve diagnostic efficiency in patients with obstructive CAD [25]. It was also shown that on-site FFR_CT_ combined with CCTA offers similar diagnostic accuracy compared to CT perfusion and CCTA [26]. Therefore, the main task for FFR_CT_ in CCTA is the evaluation not only of the anatomical significance but also of the hemodynamical relevance of a given stenosis. One approach is the application of the FFR_CT_ threshold of 0.8. However, our study results indicate that this threshold might not be applied for the evaluation of hemodynamic coronary stenosis in the distal parts of the vessels. Therefore, it is of crucial importance that CCTA and FFR_CT_ are regarded as complementary techniques instead of the isolated use of CCTA or FFR_CT_ to prevent false-positive findings.

The development and introduction of FFR_CT_ have been an important step over the last few years to not only visually grade the significance of coronary artery stenosis but also non-invasively get information about its hemodynamic and clinical relevance. Severe stenosis does not consistently result in hemodynamically relevant pressure gradients, and a significant percentage of intermediate stenosis does not even cause ischemia [27, 28] The calculation of FFR_CT_ values via post-processing of an existing CCTA dataset displayed a promising diagnostic tool without harming patients. Although many studies could demonstrate good sensitivity and specificity in comparison to invasive X-ray coronary angiography, it remains elusive if the same threshold can be applied to FFR_CT_ as for the invasive counterpart [10]. FFR_CT_ values were comparable with invasive X-ray coronary angiography values in previous studies focusing on patients with coronary stenosis [10, 19–24]. However, there is so far no data about the reliability of FFR_CT_ in apparently healthy subjects. A common finding in FFR_CT_ is the constant decline of FFR values in the more distal parts of the vessels, most probably due to the physiological tapering of the vessels. Another explanation might be the presence of endothelial dysfunction affecting the hemodynamics of the coronary arteries. However, a constant decline in more distal parts would not be generally expected in patients with endothelial dysfunction. Other influencing factors might be coronary dominance or the presence of serial lesions, as well as compensation via collaterals.

The question arises of how far distally hemodynamically significant stenosis can reliably be detected, and at which level the high diagnostic accuracy of coronary CT turns into a rate of high false-positive (FFR_CT_) findings, potentially causing harm to the patient by subsequent investigations or medication. The evaluation of the vessel diameters in participants without any coronary artery stenosis showed a significant difference in vessel size in the distal LAD and RCA depending on FFR_CT_ above or below 0.8. However, no clear cut-off can be defined.

Therefore, we propose FFR_CT_ should always be evaluated in addition to visual evaluation of the CCTA dataset so that pathologic FFR_CT_ values without any stenosis in CCTA can easily be interpreted as false positive, but probably as a sign of endothelial dysfunction. Nevertheless, in subjects with stenosis located proximal or midportion, values below 0.8 in the distal vessel might be the result of two amplifying mechanisms. Other likely reasons reported are diffuse CAD, serial lesions, small vessel size relative to myocardial mass, inadequate nitrate response, or technical misalignment [27]. This issue of falsely pathologic FFR_CT_ presumably affects a significant portion of CAD patients, since we found this effect in up to a third of our study participants without any coronary stenosis at all. Of course, clinical data and symptom presentation should also be taken into consideration. However, further studies are necessary to investigate the clinical relevance of FFR_CT_ measurements in distal vessel parts. FFR_CT_ may report pathological values so distal in the vessel that it could not be stented [29]. In this context, hemodynamically relevant stenosis also in proximal or midportion segments may be treated with initial conservative strategy in chronic coronary syndrome [30]. Decision-making based on pathological FFR_CT_ values in distal coronary artery segments should therefore be performed with the greatest caution and by a thorough consideration of visual assessment, clinical data, and patient symptoms.

### Limitations

A drawback of our study is the absence of invasive angiography FFR as a reference standard. However, in an asymptomatic cohort mostly without coronary artery stenosis, invasive angiography is not indicated and was consequently not part of the study protocol. On the other hand, this limitation might also be judged to be a strength of the study design, since routine CCTA in asymptomatic subjects with a low cardiovascular risk profile is rare. Additionally, the applied FFR software prototype in this study is not FDA-approved compared to other available applications. Due to the technical specifications of the software prototype, no side branches of the coronaries were evaluated. Furthermore, only male athletes were included in this study.

### Conclusion

Even in highly trained athletes, pathologic FFR_CT_ values ≤ 0.8 in distal coronary artery segments, suggesting hemodynamically significant stenosis, are a frequent finding, occurring in over one-third of subjects despite the absence of coronary artery stenosis. This effect is to some degree explainable by small vessel diameters and independent from coronary artery dominance. Therefore, the validity of hemodynamic relevance evaluation in distal coronary artery segment stenosis is reduced.

## Supplementary information

ESM 1 (DOCX 489 kb)

## Abbreviations

BPM: Beats per minute
CAD: Coronary artery disease
CCTA: Coronary computed tomography angiography
CTDI_vol_: Volumetric computed tomography dose index
FFR: Fractional flow reserve
FFR_CT_: Computed tomography fractional flow reserve
LAD: Left anterior descending coronary artery
LCX: Left circumflex artery
RCA: Right coronary artery

## Declarations

### Guarantor

The scientific guarantor of this publication is Patrick Krumm.

### Statistics and biometry

One of the authors has significant statistical expertise.

No complex statistical methods were necessary for this paper.

### Study subjects or cohorts overlap

Some study subjects or cohorts have been previously reported in:

Tsiflikas I, Thomas C, Fallmann C et al (2015) Prevalence of subclinical coronary artery disease in middle-aged, male marathon runners detected by cardiac CT. Rofo 187:561–568

Burgstahler C, Cipowicz H, Thomas C et al (2018) Coronary and carotid atherosclerosis in asymptomatic male marathon runners. *Scand J Med Sci Sports* 28:1397–1403

### Methodology

• Prospective

• Diagnostic or prognostic study

• performed at one institution
