# Supplementary material for: Prevalence of pathological FFRCT values without coronary artery stenosis in an asymptomatic marathon runner cohort
Source: Eur Radiol. 2021 May 26;31(12):8975–82. doi: 10.1007/s00330-021-08027-0 (PMC8589749; doi:10.1007/s00330-021-08027-0)

**Supplementary material:**

**Extended Results:**

*Overall FFR_CT_ evaluation*98 participants underwent CCTA. 90 datasets could successfully be evaluated using the FFR_CT_ prototype. Eight datasets were unevaluable for the FFR_CT_ prototype software due to prototype software coronary artery segmentation processing failure.

The detailed overview of the FFR_CT_ results is shown in Table 5. Overall, the mean FFR_CT_ value in the distal parts was 0.78 ± 0.12 in the LAD, 0.88 ± 0.08 in the LCX, and 0.88 ± 0.09 in the RCA. 35 participants (39%) showed a FFR_CT_ value below 0.8 in the LAD, eleven participants (12%) in the LCX as well as eleven participants (12%) in the RCA.

Vessel diameters between participants with FFR_CT_ values >0.80 compared to participants with FFR_CT_ values ≤0.80 were 1.6 ± 0.3 mm versus 1.5 ± 0.3 mm for the distal LAD (p=0.025), 1.8 ± 0.3 mm versus 1.6 ± 0.5 mm for the distal LCX (p=0.183), and 2.0 ± 0.4 mm versus 1.5 ± 0.2 mm for the distal RCA (p<0.001).

*Dependency of FFR_CT_ regarding stenosis groups (per-vessel segmental analysis)
LAD:*

The proximal LAD showed no significant difference between participants without stenosis compared to participants with stenosis of any degree (0.98 ± 0.02 vs. 0.97 ± 0.04; p=0.517). FFR_CT_ values were significantly different regarding the mid LAD and distal LAD between no stenosis (mid LAD: 0.93 ± 0.05; distal LAD: 0.81 ± 0.10) and stenosis of any degree (mid LAD: 0.90 ± 0.06, p=0.029; distal LAD: 0.72 ± 0.14 , p<0.001). There was no significant difference regarding vessel diameters of the LAD between the stenosis groups (Table 6).

Comparison of all three stenosis groups revealed significant differences regarding the mid LAD (p=0.049) and the distal LAD (p=0.002; Table 5; Figure 3). No significant difference was found regarding the proximal LAD (p=0.681). In the mid LAD, FFR_CT_ values below 0.8 were found in two vessels (3%) without stenosis. In the distal LAD, FFR_CT_ values below 0.8 were found in 19 vessels (31%) without stenosis.

*LCX:*

The comparison of no stenosis vs. stenosis of any degree showed no significant difference in the proximal LCX (0.98 ± 0.02 vs. 0.98 ± 0.04; p=0.627) and in the mid LCX (0.94 ± 0.06 vs. 0.91 ± 0.07; p=0.073). In the distal LCX (0.89 ± 0.07 vs. 0.80 ± 0.09) there was a significant difference between these two groups (p=0.009).

The analysis of all three stenosis groups only showed a significant difference for the distal LCX (p=0.034; Figure 4). There was no significant difference regarding vessel diameters of the LCX between the stenosis groups (Table 6). Pathological FFR_CT_ values below 0.8 without stenosis were found in the mid LCX (three vessels; 4%) and in the distal LCX (seven vessels; 8%).

*RCA:*
Comparing no stenosis vs. any stenosis, there was no significant difference in the proximal RCA (0.99 ± 0.01 vs. 0.99 ± 0.01; p=0.518), in the mid RCA (0.95 ± 0.04 vs. 0.95 ± 0.02; p=0.445), and in the distal RCA (0.88 ± 0.09 vs. 0.88 ± 0.10; p=0.741). There was also no significant difference in the comparison of the three stenosis groups (Figure 5). There was no significant difference regarding vessel diameters of the RCA between the stenosis groups (Table 6). Pathological FFR_CT_ values below 0.8 without stenosis were found in one vessel (1%) in the mid RCA and in ten vessels (12%) in the distal RCA.
Table 6 shows further details regarding vessel diameters of the stenosis groups.

**Table 5: Per vessel analysis: Computed tomography fractional flow reserve (FFR_CT_) results with number and percentage of vessels with FFR** ≤**0.8 in parentheses**

|  | **Proximal** | **Mid** | **Distal** |
| --- | --- | --- | --- |
| *Overall* |  |  |  |
| LM (n=90) | 1.00 ± 0.01 (0; 0%) |  |  |
| LAD (n=90) | 0.98 ± 0.02 (0; 0%) | 0.92 ± 0.06 (4; 4%) | 0.78 ± 0.12 (35; 39%) |
| LCX (n=90) | 0.98 ± 0.02 (0; 0%) | 0.94 ± 0.06 (4; 4%) | 0.88 ± 0.08 (11; 12%) |
| RCA (n=90) | 0.99 ± 0.01 (0; 0%) | 0.95 ± 0.04 (1; 1%) | 0.88 ± 0.09 (11; 12%) |
| *Vessels with no coronary artery stenosis* |  |  |  |
| LAD (n=62) | 0.98 ± 0.02 (0; 0%) | 0.93 ± 0.05 (2; 3%) | 0.81 ± 0.10 (19; 31%) |
| LCX (n=83) | 0.98 ± 0.02 (0; 0%) | 0.94 ± 0.06 (3; 4%) | 0.89 ± 0.07 (7; 8%) |
| RCA (n=81) | 0.99 ± 0.01 (0; 0%) | 0.95 ± 0.04 (1; 1%) | 0.88 ± 0.09 (10; 12%) |
| *Vessels with*  *non-significant coronary artery stenosis* |  |  |  |
| LAD (n=22) | 0.98 ± 0.02 (0; 0%) | 0.92 ± 0.04 (0; 0%) | 0.73 ± 0.14 (12; 55%) |
| LCX (n=5) | 0.98 ± 0.03 (0; 0%) | 0.91 ± 0.08 (1; 20%) | 0.79 ± 0.10 (3; 60%) |
| RCA (n=8) | 0.99 ± 0.01 (0; 0%) | 0.95 ± 0.02 (0; 0%) | 0.92 ± 0.03 (0; 0%) |
| *Vessels with significant coronary artery stenosis* |  |  |  |
| LAD (n=6) | 0.95 ± 0.08 (0; 0%) | 0.85 ± 0.10 (2; 33%) | 0.67 ± 0.15 (4; 67%) |
| LCX (n=2) | 0.96 ± 0.06 (0; 0%) | 0.91 ± 0.06 (0; 0%) | 0.81 ± 0.07 (1; 50%) |
| RCA (n=1) | 1.00 (0; 0%) | 0.93 (0; 0%) | 0.62 (1; 100%) |

| RCA (n=22) | 0.99 ± 0.01 (0; 0%) | 0.92 ± 0.04 (1; 5%) | 0.83 ± 0.09 (6; 27%) |
| --- | --- | --- | --- |

**Table 6: Per vessel analysis: Vessel diameters in mm**

|  | **Proximal** | **Mid** | **Distal** |
| --- | --- | --- | --- |
| *Overall* |  |  |  |
| LAD (n=90) | 3.6 ± 0.6 | 2.4 ± 0.5 | 1.6 ± 0.3 |
| LCX (n=90) | 3.6 ± 0.7 | 2.5 ± 0.6 | 1.9 ± 0.4 |
| RCA (n=90) | 4.3 ± 0.8 | 3.2 ± 0.7 | 2.0 ± 0.5 |
| *Vessels with no coronary artery stenosis* |  |  |  |
| LAD (n=62) | 3.7 ± 0.6 | 2.5 ± 0.5 | 1.6 ± 0.3 |
| LCX (n=83) | 3.7 ± 0.8 | 2.5 ± 0.5 | 1.8 ± 0.4 |
| RCA (n=81) | 4.3 ± 0.8 | 3.2 ± 0.8 | 1.9 ± 0.5 |
| *Vessels with*  *non-significant coronary artery stenosis* |  |  |  |
| LAD (n=22) | 3.6 ± 0.6 | 2.4 ± 0.3 | 1.6 ± 0.2 |
| LCX (n=5) | 3.5 ± 0.4 | 2.6 ± 0.8 | 1.8 ± 0.5 |
| RCA (n=8) | 4.1 ± 0.6 | 3.3 ± 0.5 | 2.2 ± 0.5 |
| *Vessels with significant coronary artery stenosis* |  |  |  |
| LAD (n=6) | 3.2 ± 0.6 | 2.1 ± 0.6 | 1.5 ± 0.4 |
| LCX (n=2) | 3.4 ± 0.1 | 2.7 ± 0.8 | 1.6 ± 0.1 |
| RCA (n=1) | 4.9 | 3.6 | 1.2 |

**Table 7: Influence of coronary artery dominance on pathological FFR_CT_ values ≤0.8 in participants without any stenosis (n=59).**

|  | Proximal | Mid | Distal |
| --- | --- | --- | --- |
| *Left dominant (n=7)* |  |  |  |
| LAD | 0.98 ± 0.03 (0; 0%) | 0.92 ± 0.05 (0; 0%) | 0.75 ± 0.12 (4; 57%)^a^ |
| LCX | 0.98 ± 0.04 (0; 0%) | 0.95 ± 0.06 (0; 0%) | 0.87 ± 0.08 (2; 29%)^b^ |
| RCA | 0.99 ± 0.01 (0; 0%) | 0.96 ± 0.04 (0; 0%) | 0.91 ± 0.10 (1; 14%)^c^ |
| *Right dominant (n=20)* |  |  |  |
| LAD | 0.98 ± 0.02 (0; 0%) | 0.91 ± 0.07 (2; 10%) | 0.80 ± 0.13 (7; 35%) ^a^ |
| LCX | 0.98 ± 0.02 (0; 0%) | 0.92 ± 0.07 (1; 5%) | 0.88 ± 0.08 (1; 5%) ^b^ |
| RCA | 0.99 ± 0.01 (0; 0%) | 0.97 ± 0.02 (0; 0%) | 0.91 ± 0.05 (0; 0) ^c^ |
| Codominant (n=32) |  |  |  |
| LAD | 0.99 ± 0.01 (0; 0%) | 0.94 ± 0.04 (0; 0%) | 0.82 ± 0.08 (8; 25%) ^a^ |
| LCX | 0.99 ± 0.01 (0; 0%) | 0.95 ± 0.04 (1; 3%) | 0.90 ± 0.06 (2; 6%) ^b^ |
| RCA | 0.99 ± 0.00 (0; 0%) | 0.96 ± 0.02 (0; 0) | 0.88 ± 0.07 (3; 9%) ^c^ |

Fisher-Freeman-Halton test for frequency of occurrence of pathologically low FFR_CT_ values ≤0.8 between the different groups of coronary artery dominance for each distal coronary artery respectively:

1. LAD: *p* = 0.227
2. LCX: *p* = 0.183
3. RCA: *p* = 0.170

**Supplemental Figures 3-5:**

Supplemental Figures 3-5 show FFR_CT_ evaluation of the coronaries with regard to the extent of coronary stenosis. The FFR_CT_ values decreased in all groups from proximal to distal. The mid LAD (p=0.049), the distal LAD (p=0.002), and the distal LCX (p=0.034) proved to be significantly different, depending on the degree of coronary stenosis.

*Abbreviations: FFR_CT_: CT Fractional Flow Reserve*

Figure 3
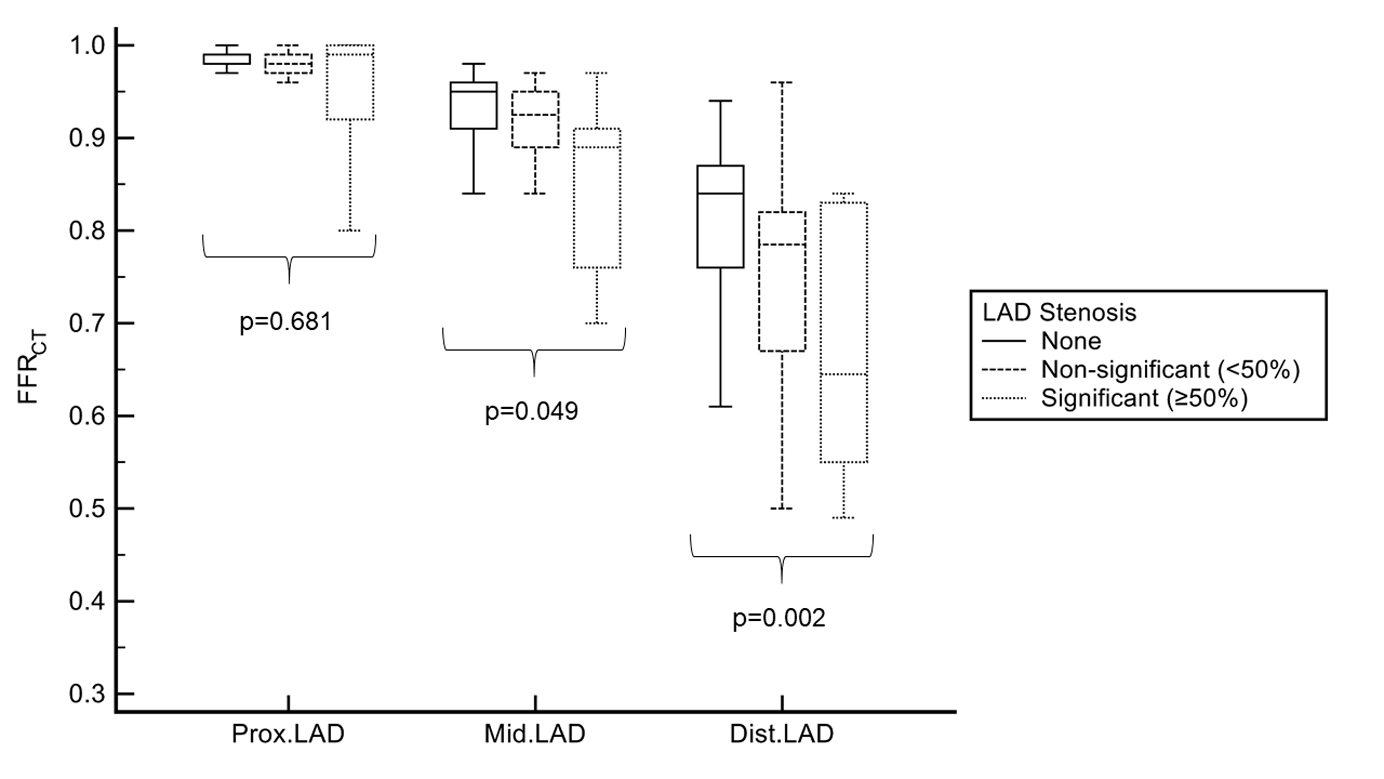


Figure 4


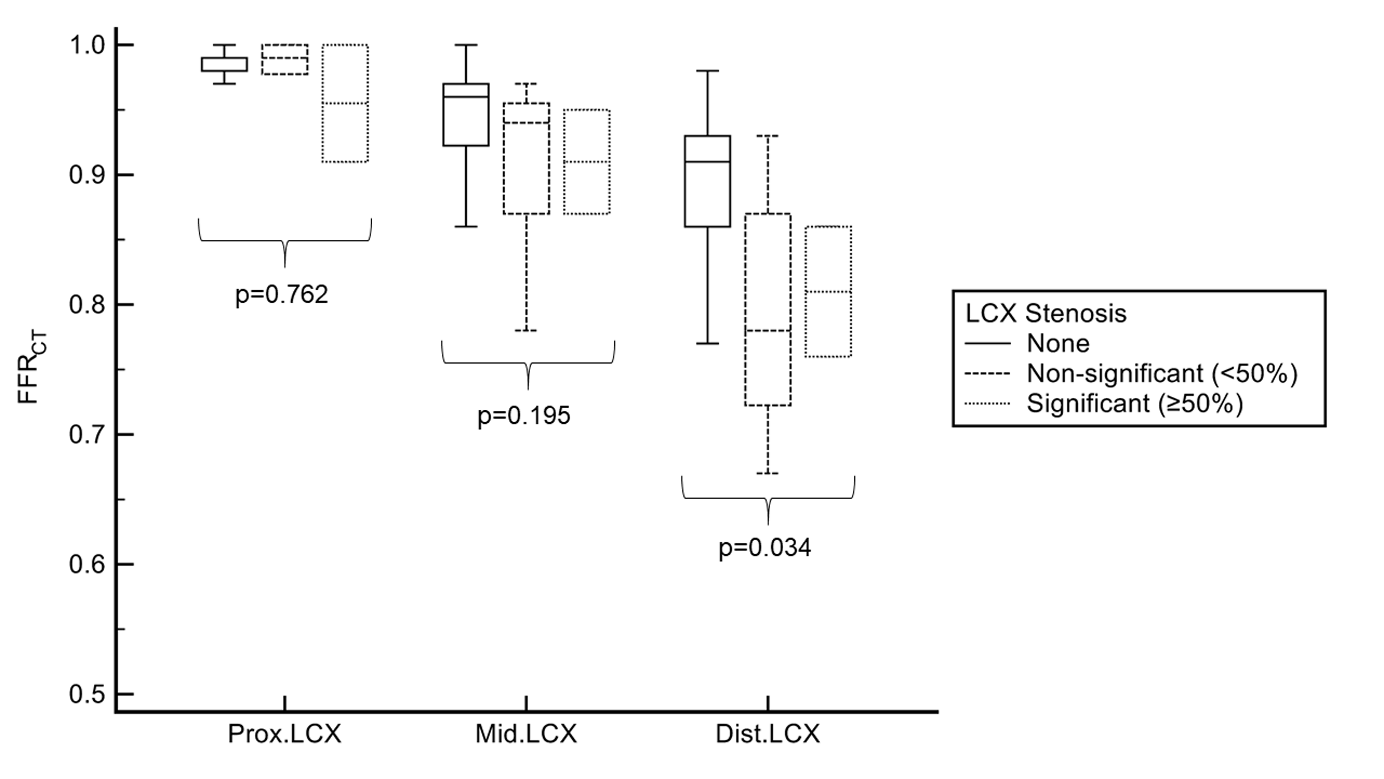


Figure 5


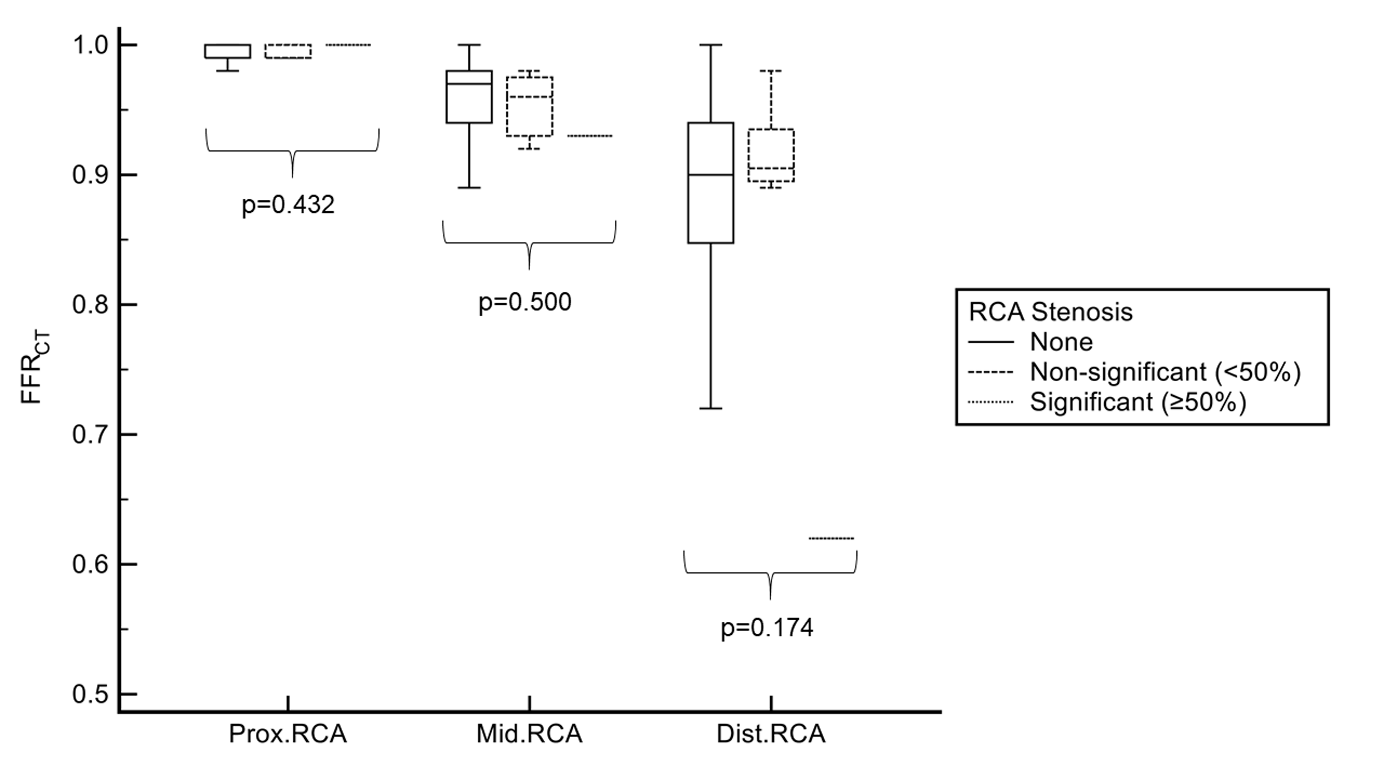

Supplement: Supplementary file 1 — (DOCX 489 kb) [file 330_2021_8027_MOESM1_ESM.docx]
